# Interactions and scattering of quantum vortices in a polariton fluid

**DOI:** 10.1038/s41467-018-03736-5

**Published:** 2018-04-13

**Authors:** Lorenzo Dominici, Ricardo Carretero-González, Antonio Gianfrate, Jesús Cuevas-Maraver, Augusto S. Rodrigues, Dimitri J. Frantzeskakis, Giovanni Lerario, Dario Ballarini, Milena De Giorgi, Giuseppe Gigli, Panayotis G. Kevrekidis, Daniele Sanvitto

**Affiliations:** 1 CNR NANOTEC, Istituto di Nanotecnologia, Via Monteroni, 73100 Lecce, Italy; 2 Nonlinear Dynamical Systems Group, Computational Sciences Research Center, and Department of Mathematics and Statistics, San Diego State University, San Diego, CA 92182-7720 USA; 3 Grupo de Física No Lineal, Departamento de Física Aplicada I, Escuela Politécnica Superior, Universidad de Sevilla, C/Virgen de África, 7, 41011 Sevilla, Spain; 4 Instituto de Matemáticas de la Universidad de Sevilla (IMUS), Edificio Celestino Mutis. Avda. Reina Mercedes s/n, 41012 Sevilla, Spain; 5 Departamento de Física e Astronomia/CFP, Faculdade de Ciências, Universidade do Porto, R. Campo Alegre, 687, 4169-007 Porto, Portugal; 6 Department of Physics, National and Kapodistrian University of Athens, Panepistimiopolis, Zografos, Athens, 15784 Greece; 7 Department of Mathematics and Statistics, University of Massachusetts, Amherst, MA 01003-4515 USA; 8 INFN Sezione di Lecce, 73100 Lecce, Italy

## Abstract

Quantum vortices, the quantized version of classical vortices, play a prominent role in superfluid and superconductor phase transitions. However, their exploration at a particle level in open quantum systems has gained considerable attention only recently. Here we study vortex pair interactions in a resonant polariton fluid created in a solid-state microcavity. By tracking the vortices on picosecond time scales, we reveal the role of nonlinearity, as well as of density and phase gradients, in driving their rotational dynamics. Such effects are also responsible for the split of composite spin–vortex molecules into elementary half-vortices, when seeding opposite vorticity between the two spinorial components. Remarkably, we also observe that vortices placed in close proximity experience a pull–push scenario leading to unusual scattering-like events that can be described by a tunable effective potential. Understanding vortex interactions can be useful in quantum hydrodynamics and in the development of vortex-based lattices, gyroscopes, and logic devices.

## Abstract (web summary)

Superfluid flow around a vortex is quantized so that vortices become discrete, particle-like defects, with interactions mediated by the surrounding fluid. Here, the authors use a polariton system to experimentally investigate the behavior and scattering of vortices in a two-component superfluid.

## Introduction

Quantum vortices^1^ correspond to wave field rotations in systems described by means of complex wavefunctions. In contrast to the classical case, the continuity of the phase constrains the orbital angular momentum (OAM, also called topological charge *l*) to be an integer number. A direct consequence is that the fluid velocity—which in a superfluid is proportional to the phase gradient—decays as 1/*r* away from the vortex core. Quantum vortices are commonly observed in a wide range of contexts, including condensates^2–6^, superconductors^7^, optics^8,9^, free electron beams^10^, and even in the recently detected gravitational waves originating from the merging of two spinning black holes^11^. In particular, quantum vortex configurations and pairing in condensates are fundamental in relation to their long-range order coherence, phase transitions, and quantum turbulence^12–14^.

Nonlinear effects enable the motion of vortices in external density and phase field gradients^15^. These interactions result in quantum vortices experiencing two main driving velocities: one parallel to phase gradients and another perpendicular to density gradients. In atomic Bose–Einstein condensates (BECs), both the case of cowinding and that of counterwinding two or few vortices have been studied^16–20^. When considering the spinor nature of two-component condensates, recent works pointed to the possible representation in terms of vortex molecules^21,22^ and to the more complex nature of the corresponding interactions^23–26^. One driving concept in such theoretical works is the perpendicular velocity exerted by the vortices on each other^27^, resulting in the orbiting/parallel motion of two co-/counterwinding vortex cores, respectively. Experimental collisional dynamics^28^, were recently induced on the timescale of seconds in the case of an antiferromagnetic spinor BEC, exploiting the counter-rotating orbits of opposite-charge vortices. The rich phenomenology of vortex dynamics observed in BECs reaches far out of their specific physical domains, including their role as cosmological simulators^29,30^. In that context, among the proposals for fundamental physical theories are schemes describing the quantum vacuum as a special superfluid/BEC medium^31–33^, and the elementary particles as quantized vortex excitations on such background^34^.

Semiconductor microcavity polaritons represent a convenient platform to achieve condensates of strongly coupled exciton and photon fields^35–37^, for the study of two-dimensional (2D) quantum hydrodynamics and topological excitations^5,6,38–42^ in dissipative and interacting superfluids. Polariton fluids are hence similar to nonlinear optics media and atomic BECs, yet possessing their own peculiarities, such as Rabi coupling^43^, nonparabolic dispersions (e.g., comprising negative mass)^44^, and strong nonlinearities^45^. One of their assets is the ability to readily generate a given initial state (setting velocity, directionality, spin and OAM, etc.); therefore, providing a full control over the quantum state of the polariton fluid^46^. Polaritons support rich spinorial patterns, in analogy to optical (multifrequency or multi-polarization) systems and multicomponent BECs^47,48^. When considering both the spin and OAM degrees of freedom, three basic vortex configurations are most relevant: the full vortex (FV), that is composed of phase singularities with the same orbital charge in both spin populations; the spin vortex (SV), which, in contrast, is composed of opposite windings between the two spin components; and the half vortex (HV), that consists of a unit charge coupled to a chargeless configuration. In the full-vortex case, the resulting polarization is spatially homogeneous, while for the other two cases a surrounding inhomogeneous texture is present. Full- and half-integer quantum vortices^49–51^ have attracted recent interest since they can be studied both under spontaneous, as well as resonant generation, and offer the possibility to shape vortex–antivortex pair-creation events^46^, vortex lattices^52,53^, and spin–vortex textures^54–56^, together with highly nonlinear dynamics^57^ and, more recently, even Rabi-vortex coupling effects^58^.

In this work we use a compact solid-state device to explore the fundamental nonlinear interactions between vortices and their dynamics in an exciton polariton fluid. We generate the polariton fluid directly embedding a pair of vortices, using a resonant pulsed excitation beam with a modified Laguerre–Gauss (LG) spatial profile. Indeed, the resonant excitation results in that the photonic pulse profile is coherently converted in a polariton fluid, representing its initial condition. In this manner we are able to seed vortices (initially locked by the resonant photonic pulse) at any desired locations and with a tunable intercore distance. The winding direction of the vortex (i.e., the OAM *l* = ±1) can be set too. Here we always initialize the fluid with a pair of cowinding vortices, inside a given spin component, in order to activate self-propelled rotations of the pair itself. After the pulsed pump completes its injection, we track the ensuing vortex dynamics for different pumping powers, which amounts to controllably varying the nonlinearity in the system. When the external pumping is increased—thus the total population and hence the effective nonlinearity—vortices are able to interact more strongly. Indeed, this increases the self-rotation effect and, in the case of a spinorial configuration with opposite-orbital charges between the two spin components, is able to split the spin–vortex states into their composing half-quantum vortices (i.e., baby-skyrmions^56^). Furthermore, contrary to the case of vortices in atomic, one component BECs, we observe that, for high enough pumping power, a radial dynamics is triggered too and the vortices start to approach each other. Such effect grants access to unexpected vortex–vortex scattering scenarios. We deploy a theoretical model to understand why vortices get closer to each other and conclude that this effect is due to strong spiral density patterns—mediated by the intrinsic excitonic nonlinearity—that channel the vortices to get closer to each other and eventually scatter. The understanding and control of vortex–vortex interactions can play a role in quantum hydrodynamics^59^, BECs phase transitions^13^, and pattern formation^60^ or superfluid vacuum theories^31^, as well as suggesting hints in the design of vortex lattices shaping^52^, ultrasensitive gyroscopes^61^, and information processing polariton devices^62^.

## Results

### A multicomponent polariton fluid

Our physical system consists of four components: the exciton and the photon fields in both the *σ*^+^ and *σ*^−^ circular polarizations (denoted as *σ* ≡ SAM = ± 1, spin angular momentum). Both spin polarizations of the photon field can be accessed (measured) independently in the experiment, while the exciton fields are not accessible for measurement. However, given that the system is only excited in one of its two normal modes, the lower polariton branch, the photon field corresponds to a one-to-one mapping of the exciton field (apart from a *π*-phase shift) and, in essence, of the polariton field. In our experiments (see the Methods section below for more details), the ultrafast imaging of the quantum fluid over tens of picoseconds reveals the in situ (2 + 1)D hydrodynamics, where the vortices can be individually tracked and their full (*x*, *y*, *t*) trajectories retrieved. This is a significant advantage over atomic BECs, where, typically, only the density can be monitored (not the phase) and where only a few snapshots from a given experimental sequence can be obtained^63^.

### Vortex dynamics

**Fig. 1:**
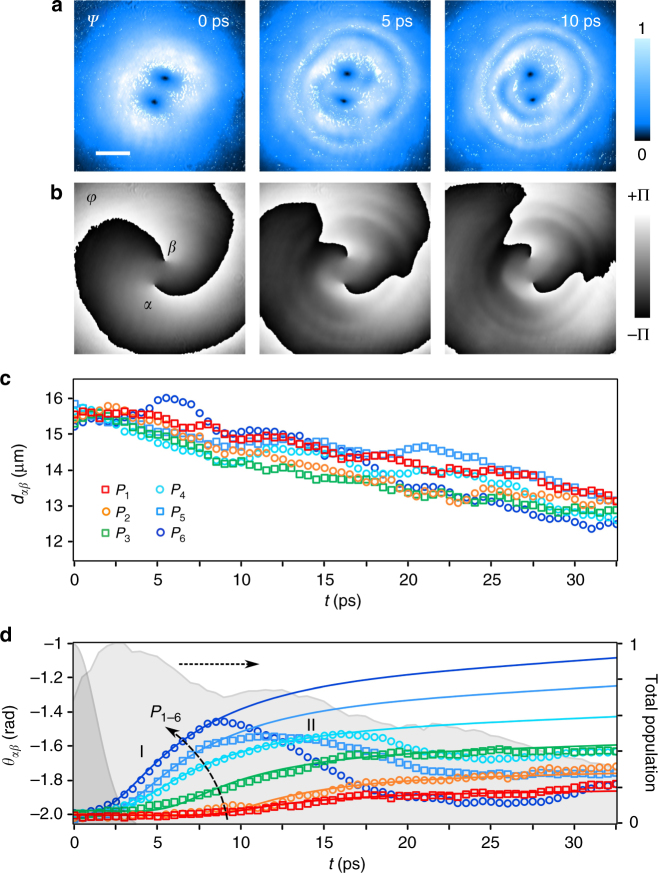
Vortex–vortex rotational pair dynamics. Polariton (**a**) amplitude and (**b**) phase maps at different times (*t* = 0, 5, and 10 ps) for the initial total polariton population *P*_6_. The two phase singularities are visible as small dark spots in **a** and labeled as *α* and *β* in **b**. Intervortex (**c**) distance and (**d**) angle for six initial total polariton populations (*P*_1_–*P*_6_). The labels I and II indicate the effective nonlinear effect and its associated rotational regime (see text). In **d** the open symbols are the experimental points and the solid lines represent fitting curves using the theoretical toy-model introduced in the Supplementary Note 6. The six pump powers correspond to the following initial populations: *P*_1–6_ ≡ 60 × 10^3^, 150 × 10^3^, 300 × 10^3^, 0.6 × 10^6^, 1.0 ×10^6^, and 1.5 × 10^6^ polaritons. The background shaded area in **d** depicts a time-series of the total polariton population plotted against the normalized *y*-axis to the right, while the darker half-Gaussian envelope on the left represents the resonant pumping temporal pulse profile. Please note that this time envelope, together with the space maps in the first frame (*t* = 0 ps) of **a**,** b** jointly represent the full spatiotemporal-phase configuration of the photonic pump pulse, setting the initial condition of the polariton fluid. The time zero (*t* = 0) of the dynamics has been chosen at its arrival, ~2.5 ps before the maximum of the total emission. The scale bar to the maps is 20 μm

The spatial structure of the vortices is reported in Fig. 1a, b, where the left panels show the photonic emission from the initial state of the polariton fluid (which bears the same spatial profile as the resonant photonic pump). The pumping density and phase distribution are the same for all the different pulse powers used here—that in turn correspond to the initial total polariton populations denoted *P*_1–6_, see Fig. 1—and, more importantly, the phase winding is the same in both spinorial components (i.e., we are initializing a pair of FV). We note that we only display in Fig. 1 the *σ*^+^ spin polarization photon field as the *σ*^−^ field is perfectly synchronized and follows, indistinguishably, the dynamics of the *σ*^+^ field (for more details please see below the section on Spinorial vortex configurations). The phase map allows for the precise determination of vortex locations (small black spots in the amplitude maps and labeled as *α* and *β* here and in the following). It is evident that the total topological charge (i.e., *l* = 2), is composed of two separated cores in the central region. The initial vortex separation, which can be controlled upon proper tuning of the optical pulse-shaping device (see Methods section below), is ~16 μm. In Fig. 1a, b we show three snapshots of the fluid at time intervals of 5 ps, for the case with the largest density (*P*_6_). Note that the vortices rotate around the center of the configuration and approximately maintain the same mutual distance, while some circular ripples are induced in the density. It is also relevant to mention that the initial size of vortex cores (as seeded by the pump) is about three times larger than the healing length *ξ* expected from the initial polariton density (*ξ* ~ 4.5 μm for *P*_1_ at 2.5 ps). However, at very long times, when the polariton population decreases significantly (population at 30 ps is about 30% of the maximum population), *ξ* should increase by less than a factor of two. Nevertheless, since the healing length represents a minimal value for the vortex core size, we can exclude any substantial effect of *ξ* on the cores size during the observed dynamics, for any of the six different power regimes (as a matter of fact, the core-size stability during the relevant time ranges can be also noted in all the amplitude maps shown here and in the following sections). On the other hand, this also confirms the strong out-of-equilibrium nature of the initial configuration and suggests that an ensuing relaxation is expected to affect the fluid reshaping and, thus, take part in mediating the intervortex rotational and radial effects described in the following. For this reason, we  characterize the observed dynamics in terms of what could be considered as effective vortex-pair interactions. A possible alternative, genuine, quasi-equilibrium regime could also be studied upon reaching thermalization (where core sizes would relax to their natural healing length) in, for example, ultra-high quality samples with lifetimes of hundreds of picoseconds^64^.

The vortex–vortex dynamics are summarized in the time plots in Fig. 1c, d which show, respectively, the intervortex distance *d*_*αβ*_(*t*) and angle *θ*_*αβ*_(*t*) in the Δ*t* = 0–32.5 ps time range (please note that here and in the following, the arrival of the photonic pulse has been chosen as the time zero of the dynamics, while the maximum polariton population is reached after 2.5 ps). The cores separation in Fig. 1c slightly decreases during the whole dynamics, with approximately the same constant speed for all the different initial densities (that is an average *v* ~ 0.04 μm ps^−1^ for each of the two vortices). We ascribe this continuous slow vortex approach to the presence of an inward phase gradient in the pump beam as it can be clearly deduced from the presence of the spiral phase patterns in Fig. 1b (see Supplementary Note 3 for more details). Such a gradient represents an external linear drive that was set upon fine tuning of the optical focusing of the pump, in order to weakly push the vortices toward each other.

### Nonlinear rotational effects

The nonlinear rotation of the vortices is evident from Fig. 1d (such nonlinear effect, termed I, along with its associated rotational regime, are present since the early times of the dynamics, the precise duration depending on power). It is crucial to note that this rotational effect is generated by the circular superfluid currents (proportional to the azimuthal phase gradients) independently generated by each vortex on the location of its partner. Furthermore, we observe that the rate of rotation of the vortices increases for stronger pump powers. This is reminiscent of the case in atomic BECs where the vortex interactions increase with the strength of the nonlinearity in the system. This can be physically interpreted by noting that increasing the density also increases the speed of sound, and thus one would expect faster motion of the vortices as they interact. In our case, as the pump power is increased, the polariton population increases and so does the effective nonlinearity—proportional to the exciton density (see Eq. (1)). At a first order approximation, this regime is expected to depend on the instantaneous and local density, which rises during the pump pulse arrival and then exponentially decays due to dissipation associated with polariton lifetime (mainly due to photon emission). The overall effect of this rise and fall of the effective nonlinearity is expected to induce a fast rotation followed by a slow deceleration in time, qualitatively resulting in an overall sigmoid shape of the *θ*_*αβ*_(*t*) curves. However, such a simplified scheme is able to capture the vortex dynamics only for short times, as shown by the solid lines in Fig. 1d, which are fitting curves using the theoretical toy-model introduced in the Supplementary Note 6. In the experiments, we observe the presence of an additional counter-rotating effect at later times for the largest powers (rotational effect and regime II, manifesting at *t* = 17, 12, and 8 ps for *P*_4,5,6_, respectively), which leads to a reversal of the rotation (rather than simply to its saturation). Here, the nonlinear reshaping of the fluid results in the formation of circular density ripples whose radial gradient represents an additional azimuthal drive on vortex motion. As the vortices ride on the inside of these (circular) radial ripples—previously reported^45,49,57^ and studied from a bifurcation perspective as ring dark (gray) solitons^65^—they provide a sharp negative radial density gradient that is responsible for the vortices rotating in a clockwise direction that overcomes the mutual vortex–vortex counter-clockwise motion. Our numerical results, implementing the generalized open-dissipative Gross–Pitaevskii (GP) model described in the Methods section (also see Supplementary Note 1), do produce the radial ripples, however, they are generated further away from the center and, therefore, they do not affect the vortex rotation (i.e., the model does not precisely reproduce the intensity or the spatial location of the rotational effect II). Nonetheless, we have checked that by changing the strength of the nonlinear interaction terms and/or the size of the pump spot width, and the initial location of the vortices, it is possible to qualitatively reproduce this rotation reversal (see Supplementary Note 2). Furthermore, our numerical simulations also reproduce the main experimental observations, including the slow vortex approach (inward drift induced by the pump radial phase), the increase of the rate of rotation as the nonlinearity increases (effect and regime I), and also corroborates the sigmoid saturation which is present in the experiment for low enough powers (i.e., the *P*_1,2,3_ cases).

### Scattering-like events

**Fig. 2:**
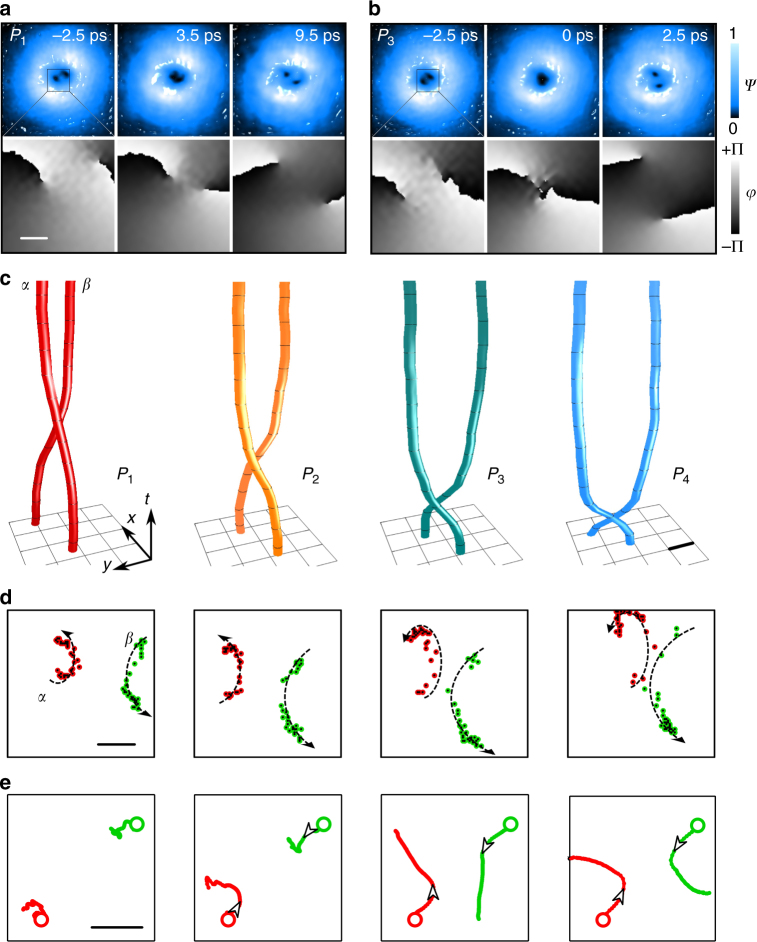
Vortex scattering. **a**,** b** Amplitude and phase maps of the polariton fluid at three time frames for a low (*P*_1_, **a**) and a larger (*P*_3_, **b**) initial density. The phase maps correspond to the small square area of the density maps. **c** The (*x*, *y*, *t*) vortex trajectories (time range Δ*t* = −2.5–9.5 ps, step *δt* = 0.5 ps) for four increasing initial populations *P*_1–4_ (the number of polaritons are reported in the caption to Fig. 3). **d** Vortex-scattering maps for the same four powers, in the Δ*t* = −2.5–13.5 ps timespan. The two arc arrows are guides for the eye to help understand their movement over time. The average and top speeds of the phase singularities are on the order of ≤1 μm ps^−1^ and ~10 μm ps^−1^, respectively. **e** (arranged in increasing pump power) Shows the numerical vortex orbits leading to scattering for large powers. Initial positions are depicted with circles. All the scale bars are 5 μm while the small square box in the top rows of **a**, **b** is 20 × 20 μm^2^

We explore the scattering between vortices after initially placing the two cores closer to each other. For this set of scattering-like experiments, we only seed vortices in the *σ*^+^ spinorial component while using a plain (vortex less) Gaussian profile in the *σ*^−^ component. We use a relative polariton population in the *σ*^−^ equivalent to ~1/4 of the polariton population in the *σ*^+^ component. We have checked in our model that changes in the relative populations between the *σ*^+^ and *σ*^−^ spinorial components do not qualitatively change the results hereby presented. In the following, we only discuss the relevant *σ*^+^ field. The corresponding experimental dynamics are shown in Fig. 2, where the initial core separation is *d*_*αβ*_ ~ 10 μm. The first two rows in the figure show the amplitude and phase maps at the initial time and successive instants, for two powers *P*_1_ (Fig. 2a) and *P*_3_ (Fig. 2b). In both cases there is a given time frame (respectively *t* = 3.5 ps and *t* = 0 ps) at which the two vortex cores appear to merge and then separate again (see right panels). When the vortices reach their closest proximity (comparable with their core radius), they cannot be clearly resolved apart in the amplitude maps. Given the access to the phase maps, where the phase singularities can be pinpointed with pixel resolution, the dynamics of the point-like entities can be tracked even when the vortex cores are nearly overlapping with each other (see also Supplementary Movies 1–4, reporting the amplitude and phase for the first four powers *P*_1,2,3,4_, respectively). The associated (*x*, *y*, *t*) trajectories extracted from the phase maps are reported in the panels of Fig. 2c, for the time range Δ*t* = −2.5–9.5 ps. The time-space vortex filaments highlight the approach and bounce back of the two cores that, after the scattering, emerge rotated compared to their initial locations. The deformation of the vortex strings in the (*x*, *y*, *t*) domain is related to the nonlinear energy stored and released by the fluid during the scattering. Nevertheless, these coherent structures robustly emerge (as individual entities) from the scattering events.

The vortex–vortex collisions are mapped in Fig. 2d, as the (*x*, *y*) trajectories for the two vortices. Upon larger initial densities, the phase singularities reach a more intimate proximity, confirming a nonlinear scattering-like process. Trajectories from the numerical simulations are reported in Fig. 2e, and qualitatively reproduce the experiments. They show how the phase singularities, slightly wandering at low power, go through stronger scattering paths upon increasing the population. We point out that in the numerical model the scattering-like events are also observed when starting with an outward phase gradient of the resonant pump, when increasing the density (see Supplementary Note 3). This is a further confirmation that the scattering events are an inherent nonlinear effect, independent from the external action of the initial pump gradient.

**Fig. 3:**
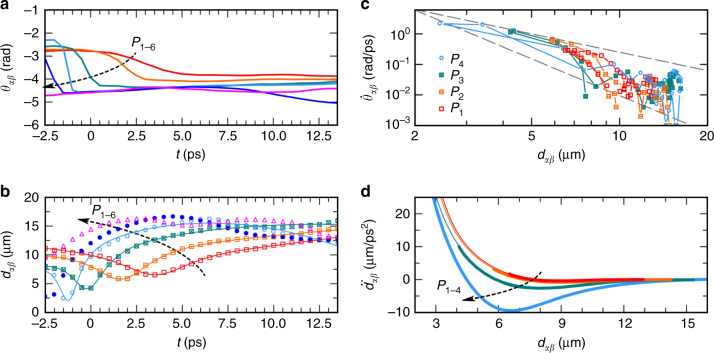
Azimuthal and radial scattering dynamics. Evolution of the intervortex (**a**) angle and (**b**) distance for different pulse powers. In **b** the dots are the experimental points and the solid lines correspond to fits using a parabola minus a Lorentzian curve *d*(*t*) = *d*_0_ + *mt* − *at*^2^ − *b*/$\left[ {\left( {t - t_{\mathrm {sc}}} \right)^2 + \sigma _{{\mathrm {sc}}}^2} \right]$ (see relevant discussion in the text). The six initial total populations correspond to *P*_1–6_ ≡ 48 × 10^3^, 96 × 10^3^, 230 × 10^3^, 460 × 10^3^, 0.96 × 10^6^, 2.4 ×10^6^ polaritons. *P*_1–4_ correspond to the dynamics reported in Fig. 2. **c** Intervortex angular velocity vs. distance in the same timespan as above in a log–log representation. The two dashed lines represent 1/*d*^2^ and 1/*d*^4^ slopes. **d** Radial acceleration $\ddot d$ vs. distance *d*. The thick lines are obtained upon differentiating the fitted *d*_*αβ*_(*t*) curves in **b** and plotted as a function of *d*_*αβ*_, while the thin lines, added for guidance, represent their $\ddot d(d)$ fits using a negative power law of *d* plus a Lorentzian well

The collisional features are recognizable in the time plots for the intervortex angle and distance in Fig. 3a, b, respectively. The angle *θ*_*αβ*_(*t*) remains approximately constant before suddenly suffering a sharp change and then settling again (sigmoid feature). The intervortex distance *d*_*αβ*_(*t*) reaches a minimum for a time in close correspondence to the inflection point of the angle *θ*_*αβ*_(*t*) curve, hence at the maximum of the angular speed, before the two vortices bounce back. We performed a fitting of the *d*_*αβ*_(*t*) curves, to retrieve an empirical trend for the collisional events upon larger excitation density. The scattering time *t*_sc_ and time width *σ*_sc_ parameters correspond to: 3.6, 1.7, −0.2, −1.2 ps and 4.7, 3.8, 2.3, 1.4 ps, respectively, for the *P*_1–4_ cases. The results for this set of experiments, and the corresponding modeling, highlight that earlier (and faster) scattering events are associated to larger powers. The rotational component during the scattering events is outlined in Fig. 3c, showing the angular velocity as a function of the separation. All the *P*_1–4_ curves represent a decreasing trend in the $\dot \theta (d)$ dependence, and lie between 1/*d*^2^ and 1/*d*^4^ power laws. It is important to contrast this observed trend in the context of point vortices in superfluid BECs. Since the tangential superfluid velocity in a BEC vortex is inversely proportional to the distance from the core, the angular velocity of a vortex pair rotating under the mutual azimuthal interaction^4,66,67^ is proportional to 1/*d*^2^. However, in our system $\dot \theta (d)$ seems to decay faster. This is attributed to the fact that the derivation of $\dot \theta \propto 1{\mathrm{/}}d^2$ assumes an effectively constant density background, while in the polariton case there is an exponentially decreasing density with time (similar results are obtained with the numerical model, see Supplementary Note 5).

### Extracting an effective potential

Finally, we show the radial acceleration during the scattering events in Fig. 3d. This plot helps to interpret the collisional dynamics as driven by an effective radial pull–push. For relatively large distances, the effective force is approximately zero leading to circular-like motion; while, at shorter ranges, the effective force acquires a negative component (and stronger for higher densities) and thus induces the vortices to get closer to each other. It is important to mention that the curves depicted in Fig. 3d are drawn under nonequilibrium conditions and cannot be straightforwardly assigned to a genuine pairwise potential between the vortices. Nonetheless, the results suggest that it is possible to induce two cowinding vortices to get closer to each other and modulate (increase) the rate of approach upon increasing the condensate’s density. Numerical simulations of our model of Eqs. (1) and (2) suggest that, while the polariton vortices drag each other in a mutual circular dance, as standard cowinding vortices do, they also induce local spiral density patterns self-channeling their approach and scattering. A cleaner depiction of this self-channeling density spiral is presented in Supplementary Note 4 (see also Supplementary Movie 5). Parametric explorations within the numerical model allow us to conclude that the vortex approach is mediated by a combination of the photonic kinetic energy term, the Rabi coupling between the photonic and excitonic components, and the intrinsic excitonic nonlinearity. The induced attraction represents a fundamental effect to be used at the basis of more complex quantum hydrodynamics and turbulence scenarios as well as in the nonlinear shaping of multicomponent vortex lattices.

### Spinorial vortex configurations

**Fig. 4:**
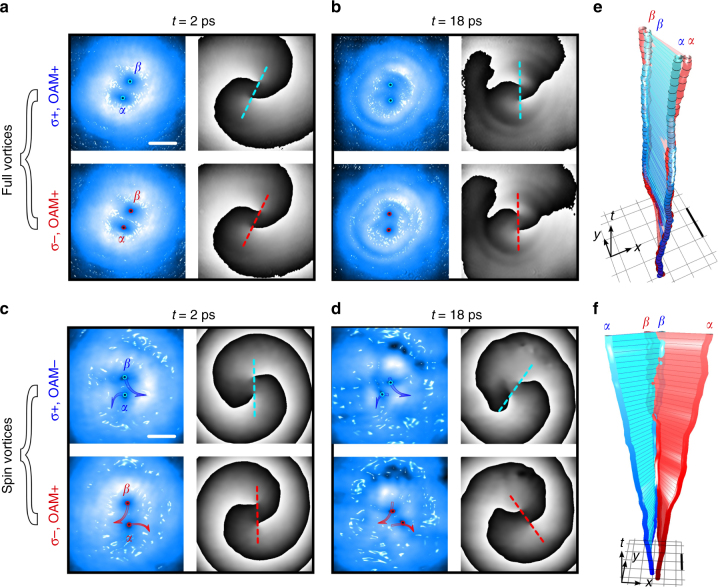
Nonlinear rotation of spinorial vortex pair configurations. **a**,** b** Amplitude and phase maps of the polariton fluid, when starting with two full vortices (same OAM in the two opposite-spin populations), at initial and later time frames. **c**,** d** Initialization with two spin vortices (opposite OAM between the two spin populations), amplitude and phase maps of the polariton fluid at different time frames. Movements of the phase singularities are tracked as blue and red dots in the amplitude maps, while the solid arrows in **c**, **d** represent the two vortex trajectories during the whole dynamics (Δ*t* = 0–35 ps). The initial total population is *P=* 450 × 10^3^ (i.e., intermediate between the *P*_3_ and *P*_4_ cases of Fig. 1). **e**,** f** Vortex (*x*, *y*, *t*) trajectories for (**e**, Δ*t* = −4–50 ps) cowinding and (**f**, Δ*t* = 0–31 ps) counterwinding cases. The scale bars are 25 μm in the space maps (**a**,** c**) and 10 μm in the (*x*, *y*, *t*) plots (**e**, **f**). The color code of labels, arrows and vortex tubes indicates the right (blue, *σ*^+^) and left (red, *σ*^−^) spin components. The dashed lines in the phase maps help highlight the opposite rotations of the vortex pairs depending on their OAM direction. Please note that the amplitude and phase maps at *t* = 2 ps (**a**,** c**) represent both the photonic pump profiles (for the two spinorial configurations) and the initial condition of the polariton fluid

The phase singularities at the vortex cores in each of the two spin populations represent elementary point-like particles with two associated quantum numbers, the *σ* and OAM (e.g., $\alpha _{{\mathrm{OAM}}}^\sigma$, where *α* represents the specific vortex core and *σ* and OAM its unitary spin and OAM charge, respectively). Depending on the direction of the quantum numbers, different vortex combinations are possible. Here it is relevant to explore the effect of the mutual azimuthal thrust on the core dynamics when seeding composite vortices with the same, as well as opposite, OAM between the two spin components (i.e., when starting with full- or spin–vortex pairs, respectively). In contrast to the results presented in Fig. 1 where both *σ*^+^ and *σ*^−^ spin polarizations remained synchronized during evolution, here the different interactions inside each of the two spin polarizations result in the two components evolving independently. We therefore need to individually measure and display the evolution in each spin component. The results are reported in Fig. 4 and validate that the rotation effect and its direction are due to the phase drive of the two vortex currents, which are present inside the same spinorial component. Indeed, in the cowinding case, the rotation is in the same direction for both the *σ*^+^ and *σ*^−^ vortex doublets, as shown in Fig. 4a, b. The initial configuration is almost identical in both spin populations albeit with a small deviation of the vortex positions between components. Despite this small initial deviation, the vortices across components stay close to each other and the overall dynamics between components stays synchronized, due to the same nonlinear effects (rotational regime I) existing in both spin populations. It is also possible that the weak attractive inter-spin interactions help in stabilizing against small differential disorder between the spin populations^57^. In contrast, for the counterwinding case, the observed trajectories (see blue and red orbits in Fig. 4c, d) are (left-to-right) mirrored across the components. The (2 + 1)D vortex lines for both co- and counter-rotating cases are reported in Fig. 4e, f, respectively. In such panels we emphasize the co- and counter-rotations by using two lines (blue and red, at any time frame) linking the *α* and *β* cores inside any of the two spin populations [and with the lines drawing two sheets in the (*x*, *y*, *t*) domain].

**Fig. 5:**
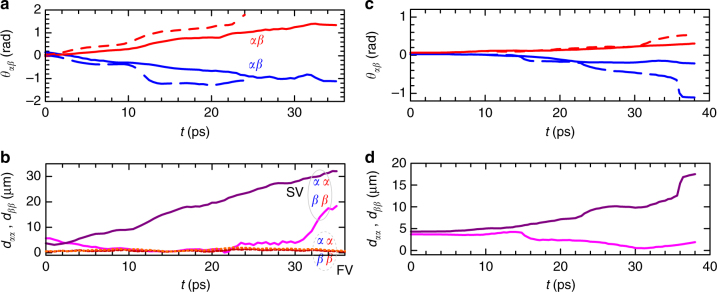
Rotational split of composite spin vortices. **a** Evolution of the intervortex angle *θ*_*αβ*_ in the spin–vortex case for the full timespan, showing the differing rotation directions for the *σ*^+^ (solid blue) and *σ*^−^ (solid red) spin components as shown in Fig. 4c, d, f. A double power case is reported too (dashed blue and red lines). **b** Inter-spin distances *d*_*αα*_ and *d*_*ββ*_ between the initially close, opposite-spin cores for both the spin vortices (SV, purple and magenta solid lines) and full vortices (FV, orange and red dashed lines). **c** Numerical intra-spin, intervortex angle *θ*_*αβ*_ and **d** the inter-spin distances *d*_*αα*_ and *d*_*ββ*_ for the counterwinding spin–vortex case, corresponding to the experimental panels **a**, **b**, respectively. The *α* and *β* labels indicate the spatially separated, cowinding, vortex cores inside each of the two spin populations, as in Fig. 4a, e, c, f

In the time plots of Fig. 5a, we confirm the opposite rotations (as solid blue and red line) in time *θ*_*αβ*_(*t*), and the faster rotation when increasing power (as dashed lines). The separation of the corresponding *α* and *β* cores between the two spins is reported in Fig. 5b (please note that such distances are labeled as *d*_*αα*_ and *d*_*ββ*_). The resulting counter-rotation of the doublets leads to the separation of at least one inter-spin couple, for which the distance increases approximately linearly (see *αα*, solid purple line). Due to slight asymmetries in the initial conditions (not a perfect alignment/tuning of the phase shaping), the second couple separation actually decreases (although, for later times, we see it separating as well, see *ββ*, solid magenta line). These observations are also exhibited by the numerical simulations that use the experimental profiles as an initial condition. In fact, the numerical plots of the intervortex angles and distance in Fig. 5c, d qualitatively reproduce those of Fig. 5a, b. In contrast, the cowinding and corotating doublets preserve the initial overlapping for the corresponding cores between the spins (the inter-spin cores distance is ~1 μm during the whole dynamics, see dashed red and orange lines in Fig. 5b).

In summary, the above set of experiments show that the vortex–vortex interactions within the same spin component dominate the dynamics, while the interactions across the components are weaker and, thus, are not essential to understand the rotational dynamics. In fact, further numerical tests, including cases varying the strength, and even eliminating, the spin–orbit coupling yielded almost identical results. Nonetheless, the two independent intra-spin rotational drives can affect the overall resulting spinorial state. For instance, topological charges seeded as full vortices (FV) keep rotating jointly. In contrast, when seeded as spin vortices (SV) they dissociate due to the effect of the opposite nonlinear rotation. Therefore, the two couples get split into four half-vortices^50,54,56,57^.

## Discussion

We have studied the external and internal effects governing the dynamics of two quantum vortices in a nonlinear and out-of-equilibrium 2D polariton fluid and discussed these in terms of effective vortex interactions. The results show that stronger density regimes enhance the effective pair interactions, accelerating or even reversing the mutual rotational effect and giving rise to unexpected radial dynamics. Indeed, nonlinearity fuels the azimuthal phase drive between vortices resulting in an increase of the speed of the circular motion for same-charge vortices. Strikingly, at short range, the intrinsic excitonic nonlinearity induces an effective radial thrust that compels the cores to approach and bounce back from each other. We exploit this feature to demonstrate unusual vortex–vortex scattering-like events. These represent an original scenario that could be further investigated in other multicomponent superfluids, and would be particularly interesting in the nonlinear shaping of vortex or vortex–antivortex lattices. More fundamental, yet intriguing, could be the case of studying the analogue dynamics when seeding opposite-charge vortex pairs (i.e., vortex–antivortex) and look for the presence and nature of a similar effective radial thrust, as well as its possible influence on pair-annihilation and nucleation events.

We further study the effects of seeding opposite-charge couples between the two spinorial components. As a result, the vortex–vortex doublets’ rotational direction in each side of the two spins is opposite, which in turn splits these composite SV into half-vortices. These structures, consisting of a unitary charge coupled to a chargeless configuration, are relevant in a wide range of fields within optical, nonlinear, atomic or high energy physics, where they are also known as Poincaré beams, vortex-bright solitons, baby-skyrmions, or filled-core vortices^4,47,68^. An interesting extension for further exploration would be to study, e.g., the interplay between two possible competing actions: the rotational split of the SV (due to the intra-spin polariton nonlinearities) and their stabilization upon the locking of the phase singularities (by the weaker inter-spin attractions). The splitting dynamics could also be investigated for the case of opposite-charge vortices which tend to move parallel to each other.

## Methods

### Experimental methods

We used a microcavity sample with an AlGaAs 2*λ* optical thickness and three 8 nm In_0.04_Ga_0.96_As quantum wells, placed in the antinodes of the cavity mode^43–45,57^. The multilayer mirrors embedding the cavity consist of AlAs/GaAs layers, with an overall photonic quality factor of *Q* = 14,000 and a resulting effective lifetime for the (lower) polariton fluid of ~25 ps, at normal incidence and at a temperature of 10 K. The quality factor of the device is associated to a photon lifetime of ~5–6 ps, resulting in a polariton lifetime of ~10–12 ps. However, time-delayed reflections back from the substrate edge help in sustaining the polariton fluid population for a longer time, resulting in the longer effective lifetime stated above. The substrate optical thickness of 1.5 mm corresponds to a 10 ps time distance of the reflected echos^45^. We performed the experiments in a defect-clean region of the sample, usually a 100 μm wide square area contained between four line dislocations. The excitation and reference beams are laser pulses with 80 MHz repetition rate and 4 ps time width (0.3 nm bandwidth). The picosecond excitation and its tuning allow the exclusive initialization of the lower polariton mode, centered at ~836.5 nm, while the upper mode that is 3 nm above (Rabi splitting of 5.4 meV) is not excited.

### Vortex generation

Double optical vortices are seeded into the system by phase shaping the plain-Gaussian LG_00_ laser pulse, into a modified LG_0±2_ state upon passage on the *q*-plate, which is a patterned liquid crystal phase retarder^57,68,69^. Indeed the initial splitting of the two unitary charges composing the LG_0±2_ state can be set upon proper tuning of the *q*-plate. Power control is set upon the use of a *λ*/2 plate and of a linear polarizer before the *q*-plate. The photonic vortex is focused at normal incidence on the sample by means of a 10 cm aspherical lens, to resonantly excite the polariton fluid. Polarization control is implemented by means of *λ*/2 and *λ*/4 plates and of linear polarizers, prior to and after the *q*-plate in order to achieve the desired combinations of topological states between the two spin populations.

### Ultrafast holographic imaging

The dynamical imaging of the polariton fluid is based on the ultrafast implementation of the so-called off-axis digital holography^43–45,57,70–72^. The sample emission is let interfere with a delayed and coherent reference beam, which is an expanded twin copy of the Gaussian excitation beam, able to provide amplitude and phase homogeneous fronts. The emission and reference beams are sent on the CCD (charge coupled device) camera with a mutual angle of inclination. This allows to obtain interferograms which are associated only to the time portion of the emission synchronous to the time arrival of the reference pulse, which can be set by a sub-micrometric step delay line. Each interferogram is analyzed by using fast Fourier transform, in the reciprocal space, where the off-axis term contains the modulation information associated to the emission at the given time. This can be filtered to retrieve the dynamics of the polariton fluid, in both amplitude and phase. The polariton phase maps are processed with a digital algorithm to retrieve all the phase singularities, whose trajectories are rebuilt. The used spatial and temporal steps here are 0.2064 μm and 0.5 ps, respectively, while the time resolution is set by the reference pulse itself to 4 ps. Every time-frame results from tens of thousands of repeated shots, which are integrated by the CCD camera set in a range between 0.15 and 1.0 ms. The visibility of the fringes remains stable for values *τ*_CCD_ ≤ 1.0 ms, which cuts out the mechanical vibrations of the setup. This procedure allows to follow deterministic vortex trajectories, while eventual stochastic (random path) vortices are washed away by the averaging of the integration process.

### Theoretical model

In the polariton literature, there have been extensive studies in both the realms of incoherent pumping^73^ and coherent coupling^74^. The present setting belongs to the latter kind, as we are resonantly pumping our sample. Therefore, to simulate our experimental setup we use a model of four coupled GP equations for the two spin (circular polarization) components for both excitons and photons:

1 $$\begin{array}{*{20}{l}} {{\mathrm{i}}\hbar \frac{{\partial \psi _ \pm }}{{\partial t}}} \hfill & = \hfill & {\left( { - \frac{{\hbar ^2}}{{2m_\psi }}\nabla ^2 - {\mathrm{i}}\frac{\hbar }{{2\tau _\psi }}} \right)\psi _ \pm + \frac{{\hbar {\mathrm{\Omega }}_{\mathrm{R}}}}{2}\phi _ \pm } \hfill \\ {} \hfill & {} \hfill & { + g_{11}\left| {\psi _ \pm } \right|^2\psi _ \pm + g_{12}\left| {\psi _ \mp } \right|^2\psi _ \pm ,} \hfill \end{array}$$

2 $$\begin{array}{*{20}{l}} {{{i}}\hbar \frac{{\partial \phi _ \pm }}{{\partial t}}} \hfill & = \hfill & {\left( { - \frac{{\hbar ^2}}{{2m_\phi }}\nabla ^2 - {{i}}\frac{\hbar }{{2\tau _\phi }}} \right)\phi _ \pm + \frac{{\hbar {\mathrm{\Omega }}_{\mathrm{R}}}}{2}\psi _ \pm } \hfill \\ {} \hfill & {} \hfill & { + \chi \left( {\frac{\partial }{{\partial x}} \pm {{i}}\frac{\partial }{{\partial y}}} \right)^2\phi _ \mp + F_ \pm ,} \hfill \end{array}$$

where *ψ*_±_ and *ϕ*_±_ represent, respectively, the wavefunctions for the excitons and photons (± indicates the two polarizations), *m*_*ψ*_ and *m*_*ϕ*_ are the respective masses and *τ*_*ψ*_ and *τ*_*ϕ*_ are the respective lifetimes, Ω_R_ is the Rabi coupling frequency, *g*_11_ is the intra-spin exciton–exciton interaction strength, while *g*_12_ represents the inter-spin interaction strength, *χ* is the coefficient of the spin–orbit coupling, and *F*_±_ is the applied external laser pulse. The value for the Rabi splitting is taken to be Ω_R_ = 5.4 meV and the exciton–exciton interaction strength^45^ *g*_11_ = 2.0 μeV μm^2^. The exciton and photon lifetimes relevant for this experiment are *τ*_*ψ*_ = 1000 ps and *τ*_*ϕ*_ = 5 ps, respectively. We consider the strength of the inter-spin exciton interaction to be an order of magnitude weaker than the intra-spin interaction, so that *g*_12_ = −0.1*g*_11_. The photon mass *m*_*ϕ*_ = 3.6 × 10^−5^*m*_e_, where *m*_e_ is the electron mass, was extracted from the dispersion relationship. On the other hand, the exciton mass is approximately four orders of magnitude larger than the photonic one, so that the exciton kinetic term could be in principle safely neglected. Also, we treat the mass of the transverse electric component of the cavity mode, $m_\phi ^{{\mathrm{TE}}}$ as being around 95% that of the transverse magnetic component $m_\phi ^{{\mathrm{TE}}}$(≈5 × 10^−5^*m*_e_), so that the strength of the TE–TM splitting is taken to be $\chi = \frac{{\hbar ^2}}{4}\left( {\frac{1}{{m_\phi ^{{\mathrm{TE}}}}} - \frac{1}{{m_\phi ^{{\mathrm{TM}}}}}} \right)$ = $0.019 \times \frac{{\hbar ^2}}{{2m_\phi }}$. Finally, the external laser pulse is modeled as a coherent pump term in the photon field of Eq. (2), by writing

3 $$F_ \pm ({\bf{r}},t) = f_ \pm \times R_ \pm ({\bf{r}}) \times T(t),$$

where for the spatial part *R*_±_ we used the normalized experimentally measured 2D spatial profiles—corresponding to a modified LG profile with the appropriate number of vortices that are seeded in the condensate—and for the temporal part *T*(*t*) we used

4 $$T(t) = {\mathrm{e}}^{ - \frac{{\left( {t - t_{\mathrm{0}}} \right)^2}}{{2\sigma _t^2}}}.$$

The strength of the laser pulse, *f*_±_, is chosen so as to replicate the observed total photon output in the experiments. The duration of the probe *σ*_*t*_ was chosen in order to correspond to a FWHM_*t*_ = 4 ps, in line with the experimental realization. The pump is instantiated some time into the simulation, reaching its maximum at *t*_0_ = 5.5 ps and removed completely after 5*σ*_*t*_ so as to negate any unintended phase locking.

### Data availability

The data that support the findings of this study are available upon request from the corresponding author.

## Electronic supplementary material

Supplementary Information(PDF 2861 kb)

Description of Additional Supplementary Files(PDF 246 kb)

Supplementary Movie 1

Supplementary Movie 2

Supplementary Movie 3

Supplementary Movie 4

Supplementary Movie 5
