# Supplementary material for: Interactions and scattering of quantum vortices in a polariton fluid
Source: Nat Commun. 2018 Apr 13;9:1467. doi: 10.1038/s41467-018-03736-5 (PMC5899148; doi:10.1038/s41467-018-03736-5)
Supplement: Supplementary file 1 — Supplementary Information(PDF 2861 kb) [file 41467_2018_3736_MOESM1_ESM.pdf]

SUPPLEMENTARY INFORMATION:
Interactions and scattering of quantum vortices in a polariton fluid

Supplementary Note 1: Model implementation and experimental data assimilation

For our numerics we rescale space using $(x, y) = \xi \times (X, Y)$ where $\xi = \sqrt{\hbar/(m_\phi \nu)}$ and time using $\tau = \nu \times t$ where ν is an arbitrary frequency chosen such that one unit of computational time τ is equivalent to one ps in the original system. Using these rescalings, the equations of motion in rescaled space (X, Y) and time τ yield:

$$\mathrm{i}\frac{\partial \tilde{\psi}_\pm}{\partial \tau} = \left(-\frac{\epsilon}{2}\nabla^2 - \mathrm{i}\gamma_\phi + |\psi_\pm|^2 + \bar{g}_{12}|\psi_\mp|^2\right)\psi_\pm + \omega_\mathrm{R}\,\tilde{\phi}_\pm \quad (1)$$

$$\mathrm{i}\frac{\partial \tilde{\phi}_\pm}{\partial \tau} = \left(-\frac{1}{2}\nabla^2 - \mathrm{i}\gamma_\psi\right)\phi_\pm + \omega_\mathrm{R}\,\tilde{\psi}_\pm + \tilde{\chi}\left(\frac{\partial}{\partial X} \pm \mathrm{i}\frac{\partial}{\partial Y}\right)^2 \tilde{\phi}_\mp + \tilde{F}_\pm, \quad (2)$$

where $\bar{g}_{12} = -0.1$, $\gamma_\psi = 0.001$, $\gamma_\phi = 0.2$, $\omega_\mathrm{R} = 4.1$, $\tilde{\chi} = 0.0128$, $\epsilon = 10^{-4}$, $\xi = 1.5415$ µm, and $\nu = 10^{12}$ s^{-1}.

In order to be able to manipulate the phase of the pump for the numerical computations, we employed a standard nonlinear least squares fitting routine using the following ansatz for the phase portion:

$$\varphi(x_i, y_i, a_i, b_i, \varphi_0) = \theta_0 + \theta_1 + \theta_2 + \varphi_0, \quad (3)$$

where φ_0 is a constant phase (shift) ansatz parameter and the θ_i contributions are given by:

$$\theta_0 = a_0\,R_0 + b_0\,R_0^2, \quad (4)$$

$$\theta_1 = a_1\,R_1 + b_1\,R_1^2 + S_1\,w_1, \quad (5)$$

$$\theta_2 = a_2\,R_2 + b_2\,R_2^2 + S_2\,w_2, \quad (6)$$

where S_i are the vortex charges, w_i and (R_1, R_2) are the angles and distances measured from the centers of the vortices (x_i, y_i) which in turn are ansatz parameters. The ansatz parameters a_i and b_i provide the linear and quadratic coefficients for the radial dependence on the phase of the fit. The distance R_0 is measured from the point (x_0, y_0), another fitting parameter, that accounts for the center of the main radial gradient of phase that, in turn, provides the inward (or outward, after a suitable modification) current flow generated by the pump. Once a pump profile is given from the experiment (modulus and phase), we apply a standard nonlinear least squares fitting algorithm to find the best parameter combination. It is worth mentioning that in order to get convergence, the fitting procedure had to be started with a judicious choice of initial parameters that were relatively close to the desired solution. Furthermore, the cost function was designed to get rid of the phase discontinuity of φ (after writing it modulo 2π) by constructing the continuous phase function $\bar{\varphi} = \varphi \times [\varphi \le \pi] + (2\pi - \varphi) \times [\varphi > \pi]$ where $[q]$ denotes the boolean operation 1 if q is true and 0 otherwise.

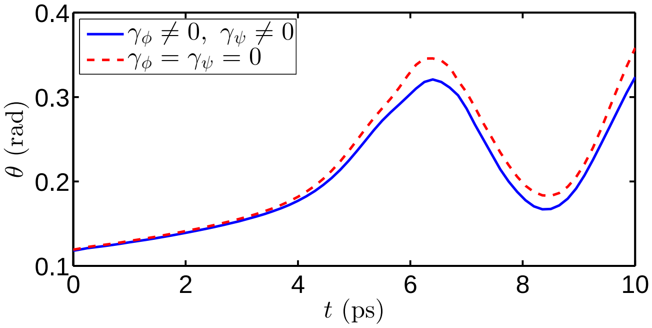

Supplementary Fig. 1: **Reproducing the rotation inversion.** Mutual angle as a function of time for a tight ($\sigma = 4$ µm) Gaussian pump. The solid (blue) and dashed (red) lines correspond, respectively, to the cases without and with dissipation. For short times ($t < 6$ ps) the dynamics is dominated by the counter-clockwise rotation induced by vortex-vortex interaction. However, the emergence of circular ripples in the background density is responsible for a rotational reversal of the vortices starting at 6 ps. After the circular ripples expand towards the periphery of the density ($t > 8$ ps), the vortices are left to continue their mutual interactions in the counter-clockwise direction.

Supplementary Note 2: Vortex rotation inversion

As noted in the main text, for large pump powers (see P_{4-6} in Fig. 1 of the main text), the vortices experience rotation reversal —namely a rotation in the opposite direction to the vortex-vortex interaction. We ascribe this unexpected rotation inversion to the gradual emergence of (circular) radial density ripples as reported in Refs. [1–4]. As the vortices ride on the inside of the circular ripples, the radial density gradient induces a rotational velocity that is in the clockwise direction, namely, in the opposite direction of the (counter-clockwise) vortex-vortex interaction. Therefore, for large enough powers, and thus deep enough circular density ripples, the local density gradient experienced by the vortices is strong enough to slow down and even overcome the mutual vortex-vortex rotation. As a consequence, these vortices are observed to change the rotational direction for large pump powers. Our numerical model implemented with the parameter values described in the previous section produce the radial ripples at a distance larger than the location of the

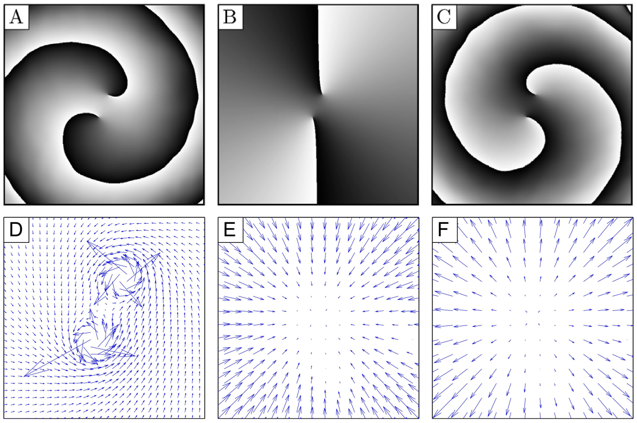

Supplementary Fig. 2: **Manipulation of the phase and flow for the pump corresponding to a same-charge vortex pair.** Phase maps (top line of panels) are shown in a 120×120 microns window while the flow maps (bottom line of panels) are shown in a smaller 40×40 microns window. (A) The original phase retrieved from the experiment. After spatially fitting the strength and phase of the pump, it is possible to only keep the contribution due to the vortex pair [see panel B] or to revert the sign of the background phase (namely, the phase not coming from the vortex pair) [see panel C] to change the direction of the background flow without altering the charge of the vortices. (B) Corresponding flow map to the full pump depicted in panel A. (E) After removing the contribution from the vortex pair the flow map clearly shows an inward flow. (F) Outward flow corresponding to the background phase depicted in panel C.

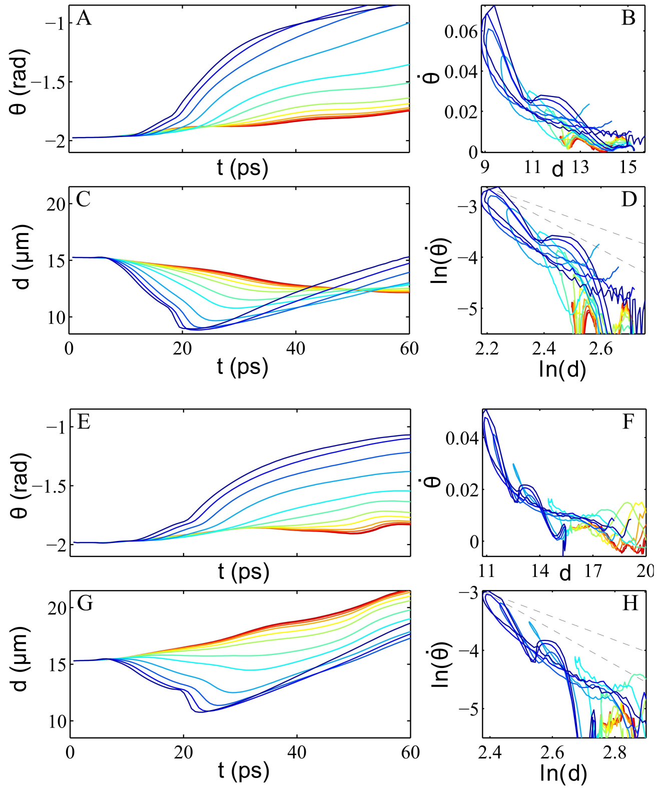

Supplementary Fig. 3: **Orbits for a same-charge vortex pair.** Mutual angle (A) and distance (C) as a function of time. The red-to-blue hues correspond to increasingly strong pump powers. All angles and distances are measured, respectively, in radians and microns. Angle vs. distance in linear (B) and log-log scales (D). For guidance, panels D and H also show, using thin dashed lines, slopes corresponding to the power laws $\dot{\theta} \propto d^{-2}$ and $\dot{\theta} \propto d^{-3}$. Panels A–D correspond to a pump with an inward flow (the one use in the experiments) while panels E–H correspond to a pump with an outward flow. Note that despite the pump flow pushing vortices outward in panel (G), for large pump powers, the vortices still get closer to each other for intermediate times.

vortices and thus missing the above-mentioned vortex rotation inversion. Nonetheless, by modifying the model parameters —specifically by making the pump spot narrower and appropriately choosing the initial location of the vortices— our model is able to reproduce the vortex rotation reversal as depicted in Supplementary Fig. 1. We have checked that the rotation inversion is only affected weakly by changes in the dissipation (contrast the two curves depicted in Supplementary Fig. 1).

Supplementary Note 3: Slow inward drift induced by pump imprinting

Using the phase fitting algorithm described in the Supplementary Note 1 we are able to control the different (e.g., radial and azimuthal) contributions to the phase of the pump. For instance, starting with the phase distribution in Supplementary Fig. 2A, we can extract the contribution of the (essentially azimuthal) vortex pair field contained in it [see Supplementary Fig. 2B]. It is important to stress that the phase profile contains all the velocity flows imparted by the pump. Specifically, the gradient of the phase in the condensate can be associated with the fluid velocity. In panel D of Supplementary Fig. 2 we depict the velocity flow associated with the experimental phase pump of panel A. Now, by removing the contribution due to the vortices from the phase of the pump, it is possible to extract an approximately radial phase gradient that is responsible for an inward flow as depicted in panel E. It is this inward flow —referred to as an external effect in the main text— that is responsible for the initial movement of the vortices, particularly at low pump powers (i.e., weak nonlinearity) where the vortices interact weakly.

In fact, in order to qualitatively pinpoint the contribution due to this inward flow on the vortex interactions/trajectories, we designed an alternative phase pump with the same two vortices but the *opposite* radial phase gradient [see panel C] which gives rise to an outward flow [see panel F]. The results from the simulations using the original inward flow and the modified outward flow are depicted, respectively, in the top and bottom

quartet of panels in Supplementary Fig. 3. The respective left columns depict the vortex-vortex angle (top subpanels) and distance (bottom subpanels) as a function of time. Note that the numerics confirm the experimental observation that the intervortex distance initially decreases [see panel C] due to the inward flow provided by the pump. However, at later times and, more strongly so, for stronger pump powers, the vortices tend to approach each other (the origin of this unusual behavior is explained in the next section) leading to scattering-like events. It is crucial to investigate if the inward flow is solely responsible for these scattering events. It is precisely for such purpose that we reverse-engineered all the different contributions of the experimental phase profiles in order to obtain those of Supplementary Fig. 2C. By doing so, while preserving (i) the same density profile, (ii) the vortex locations and (iii) their charges, we are able to revert the inward flow to an outward one. The resulting intervortex distance is depicted in Supplementary Fig. 3G. As it is clear from the figure, the outward flow is indeed responsible for pushing the vortices outward (at least for weak pumping powers). However, as the pump power is raised, surprisingly, the vortices again tend to approach each other leading to scattering collisions. Therefore, we conclude that the inward flow provided by the pump radial phase gradient is responsible for the weakly approaching dynamics observed in Fig. 1 of the main text and, in turn, helps to initiate the scattering-like collision events. Nonetheless, more importantly, there is another (nonlinear) process that is chiefly responsible for pushing the vortices closer to each other and thus leading to their scattering dynamics. It is this effect that we are now interested in describing.

Supplementary Note 4: Vortex-vortex apparent attraction

When describing the origin of vortex motion in condensates it is crucial to realize that the motion that a vortex experiences when embedded in a complex background is due to two, approximately independent, velocity contributions: [5]

(i) a velocity in the direction of the background's phase gradient and

(ii) a velocity that is perpendicular to density gradient of the background.

Therefore, not only phase gradients but also density gradients of the background drive the vortex dynamics. When two vortices are placed sufficiently far apart from each other (a few healing lengths away) in an approximately flat-density background, the dominant contribution to the motion of each vortex is the azimuthal phase gradient introduced by the presence of the other vortex (rotational effect of regime I described in the main text). However, in our case, as we now show, sizeable

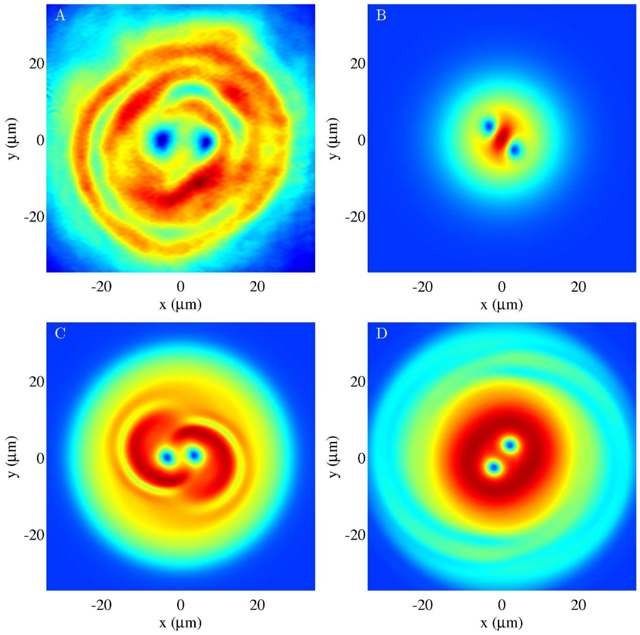

Supplementary Fig. 4: **Understanding the effective attraction between vortices.** (A) Experimental image of the density for P_6 in Fig. 1 of the main text depicting the spiral gradients in density at $t = 19$ ps. (B–D) Snapshots for the density computed by our model corresponding to the following times: (B) at maximum pump power ($t = 5$ ps), (C) during rapid decrease of inter-vortex distance ($t = 12.5$ ps), and (D) at minimum inter-vortex distance ($t = 22$ ps). The whole sequence is reported in the Supplementary Movie 5.

density gradients are also present and need to be also considered when vortices are close to each other. In fact, the approaching of the vortices described previously is a consequence of the background density gradients acquiring a spiral shape. See for instance Supplementary Fig. 4A where the snapshot of the experimental density at $t = 19$ ps is depicted. In this plot, the density gradients seem to lose their initial symmetry and they appear as spiral-like due to the rise of an azimuthal component in the density gradient. The emergence of this effect can be understood by closely following the dynamics in the Gross-Pitaevskii (GP) model. The system is pumped with two vortices embedded within a Gaussian background. The resulting dynamics are depicted in panels B–D in Supplementary Fig. 4. Panel B correspond to $t = 5$ ps when the pump is about to reach its maximum strength and the condensate is locked by the pump. It is clear from this panel that the resulting density contains a small hump at the center that is created by the inward flow provided by the radial phase gradient of the pump (see above). However, more importantly, although the vortices cannot perform a rotation around the center because their positions are locked by the forcing pump, they induce a state where the central hump has a "twist" due to the flow induced by the vortices (through their phases). Upon release of the pump, the vortices follow a

combination of the following regimes: (a) the inward (or outward) push due to the initial pump radial phase gradient remaining in the background, (b) the vortex-vortex rotation due to their phases, and (c) a velocity that is perpendicular to the density gradient of the "main" central hump and other background density gradients. It is the last ingredient (being not purely radial) that starts accelerating the inward motion of the vortices. A few picoseconds later, the central hump is replaced by density spirals due to the intrinsic rotation between vortices [see panel C]. These spiral gradients create a guiding "channel" for the vortices and effectively produce an inward pull that is responsible for the observed inward motion of the vortices leading to the scattering-like events. Performing an exhaustive parametric search controlling the different terms in Supplementary Eqs. (2) and (3), we have concluded that the *minimal model* that can reproduce the effective "attraction" between vortices contains (i) the photonic kinetic energy term, (ii) the intrinsic excitonic nonlinear term, and (iii) the Rabi coupling between the exciton and photon fields. The combination of these terms is responsible for the spiral channelling leading to the scattering-like events. In fact, we have verified that altering the relative masses between the two components ($\epsilon = 1$ or $\epsilon = 0$), eliminating losses ($\gamma_\psi = \gamma_\phi = 0$), removing the inter-spin nonlinear interaction ($g_{12} = 0$), and/or removing the spinor-coupling term ($\chi = 0$) leads to the same qualitative phenomenology of the spiraling channels.

Finally, after the vortices have scattered from each other, the background density gradient retakes an approximate radial shape [see panel D in Supplementary Fig. 4] and it thus no longer contributes to the radial motion of the vortices. After this scattering-like event, vortices drift outwards due to the dissipative terms in the system while their angular rotation slows down as the vortices are further apart from each other and that the overall density is decreasing (and so is the nonlinearity responsible for vortex-vortex motion).

Supplementary Note 5: Vortex scattering

As described in the main text, as the vortices experience an apparent effective attraction, they eventually collide in a scattering-type event. We emulate the experimental vortex dynamics depicted in Fig. 2 of the main text using our GP model. The results, using the same conditions as in the experiment, are depicted in Supplementary Fig. 5 and qualitatively match the experimental observations. In particular, the panels A and C, reporting the intervortex angle and distance over time, respectively, confirm the scattering events and the collisions to be faster for larger populations (i.e., larger nonlinearities). Furthermore, the angular velocity plotted in panels B and D validates that the vortex-vortex interaction is highly nonlinear and corresponds to a faster

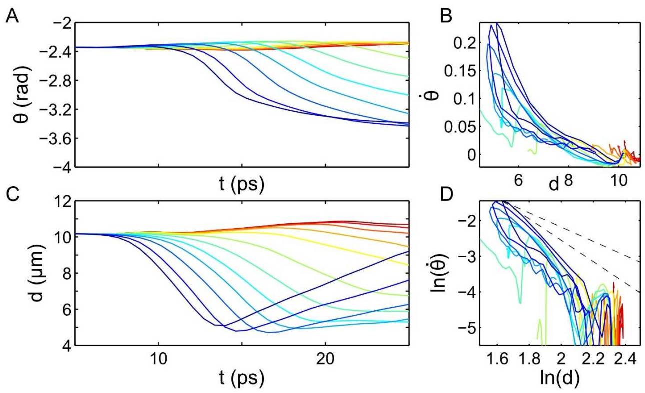

Supplementary Fig. 5: **Vortex scattering.** Numerical vortex scattering corresponding to the experimental vortex scattering of Fig. 2 of the main text. Intervortex angle (A) and distance (C) as a function of time. The red-to-blue hues correspond to increasingly strong pump powers. Angular speed vs. distance in linear (B) and log-log scales (D). For guidance, panel D also shows, using thin dashed lines, slopes corresponding to the power laws $\dot{\theta}/d^2$ and $\dot{\theta}/d^3$. The setup is the same as in Fig. 1 of the main text but for vortices starting closer to each other.

power law than for standard vortices in a superfluid with $\dot{\theta} \propto 1/d^2$. In fact, the log-log plot depicted in Supplementary Fig. 5D shows that the $\dot{\theta}$ vs. d curves lie below $1/d^2$ and $1/d^3$ as in the experimental data depicted in Fig. 3 of the main text. This again suggests that the faster decay of the angular velocity when compared to an atomic BEC might be attributed to the exponential decrease in density that in turn weakens the vortex-vortex interaction.

Supplementary Note 6: Toy-model for the vortex-vortex rotation

Finally, let us understand the saturation of the vortex rotation as the overall density decreases (i.e., saturation of the rotation, as seen from the sigmoid shapes of the Fig. 1 of the main text). In a Hamiltonian (atomic) BEC model, we expect that the vortex-vortex interaction through their phase fields results in a tangential drive depending monotonically on the local and instantaneous density $\rho(x, y, t)$ (polariton population) [6, 7]. The quantized phase winding of each single vortex is equal to 2π along any path around the vortex. Hence the associated azimuthal wavevector is equal to $k = 2\pi/(2\pi \cdot d)$ with d the distance from the core. Such azimuthal k represents the *linear* speed of the induced rotation. Therefore, the *angular* speed for two well-separated vortices —i.e. with core sizes, or healing length, smaller than their mutual separation so that they chiefly interact through their phases— of equal charge, asymmetrically placed from the

center, can be described by:

$$\dot{\theta} = \frac{f(\rho)}{d^2} \tag{7}$$

where θ is the intervortex angle, d is the intervortex separation, and f is an increasing function such that $f \to 0$ when $\rho \to 0$ (as vortices do not interact in the linear limit). To simplify our exposition, let us assume that our system operates in a regime of moderate densities for which one could take the first order approximation for f as $f(\rho) = B\,\rho$ where B is a constant depending on the physical parameters of the system. If we now assume that the temporal and spatial variations of the density are slow, the main contribution to the vortex-vortex interaction will be mediated by the *local*, in space and time, density where the vortices are embedded. Thus, we arrive at the following adiabatic toy-model for the angular speed between same-charge vortices:

$$\dot{\theta}(t) = B\,\frac{\rho(r_0, t)}{d^2} \tag{8}$$

where $\rho(r_0, t)$ is the background density at a distance r_0 from the center at time t. Now, let us write a differential equation for the time behavior of the local density as the pump is raised and decreased. As in the experiments, we consider a Gaussian (in time) pulse pump with power A (this maximum pulse power occurs at time t_0) and time width q. As we are operating in a regime where the vortex core size is small compared to the size of the background density cloud, it is reasonable, for this coarse vortex-vortex interaction model, to neglect spatial variations on the background density in the vicinity of the vortex location. Doing so, and incorporating the dissipation rate γ of the lower polariton (LP) branch, yields the following approximate evolution equation for the local (constant in space) density:

$$\dot{\rho}(t) = A\,\exp\left(-\frac{(t-t_0)^2}{q^2}\right) - \gamma\,\rho(t). \tag{9}$$

Upon double time integration of Supplementary Eqs. (8) and (9), we obtain the following functional form for the inter-vortex angle dynamics:

$$\begin{aligned}\theta(t) = \theta_0 + \frac{qAB\sqrt{\pi}}{2\gamma d^2}\Bigg\{&\mathrm{erf}\left(\frac{t_0}{q}\right) + \mathrm{erf}\left(\frac{t-t_0}{q}\right) + \\ &+\left[\mathrm{erf}\left(\frac{\gamma q}{2} - \frac{t-t_0}{q}\right) - \mathrm{erf}\left(\frac{\gamma q}{2} + \frac{t_0}{q}\right)\right] \\ &\times\exp\left(\frac{\gamma q}{4} - \frac{t-t_0}{q}\right)\gamma q\Bigg\},\end{aligned} \tag{10}$$

which corresponds to a saturation of the angle as $t \to \infty$. As explained above, a couple of approximations were used in order to obtain this toy model for vortex-vortex interactions. Our goal is to understand from a *qualitative* level some of the dynamics observed in the experiments. In particular, we are interested in describing the saturation effects of the rotation that are observed (mainly at low densities) in Fig. 1 of the main text. For that purpose we now consider, for simplicity, the inter-vortex distance, as seen in the experimental dynamics, to follow the linear relation $d = d_0 - 2vt$. It is important to mention that this linear dependence for $d(t)$ is not considered in the derivation of Supplementary Eq. (10) but only used during the fitting of the experimental data of the inter-vortex angle $\theta(t)$. We use, as measured in the experiments, $d_0 = 15.6$ µm and $v = 0.04$ µm/ps. The parameters A and B were used together as a single fitting parameter. The parameters q and t_0 represent the pulse width (set to 2 ps) and central time, respectively, while γ is the decay rate of the LP branch ($\gamma = 1/\tau \sim 0.2$ ps^{-1}). The free parameters in the fit were only θ_0, t_0 and AB. The results of the fitting procedure, depicted in Fig. 1D of the main text, show that the above toy-model is able to capture well the saturation of the rotation dynamics for vortex-vortex interactions (specially for low densities). The retrieved coefficients for the pump strengths are: $AB = 0.96$, 1.58, 2.56, 3.65, 5.08, 6.22, that correspond to the experimental populations: $P_{1-6} \equiv 60 \cdot 10^3$, $150{\cdot}10^3$, $300{\cdot}10^3$, $0.6{\cdot}10^6$, $1.0{\cdot}10^6$, and $1.5{\cdot}10^6$ polaritons.

Supplementary References

[1] Liew, T. C. H., Kavokin, A. V. & Shelykh, I. A. Excitation of vortices in semiconductor microcavities. *Phys. Rev. B* **75**, 241301(R) (2007).

[2] Dominici, L. *et al.* Real-space collapse of a polariton condensate. *Nat. Commun.* **6**, 8993 (2015).

[3] Dominici, L. *et al.* Vortex and half-vortex dynamics in a nonlinear spinor quantum fluid. *Sci. Adv.* **1**, e1500807 (2015).

[4] Rodrigues, A. S. *et al.* From nodeless clouds and vortices to gray ring solitons and symmetry-broken states in two-dimensional polariton condensates. *J. Phys.: Condens. Matter* **26**, 155801 (2014).

[5] Kivshar, Y. S., Christou, J., Tikhonenko, V., Luther-Davies, B. & Pismen, L. M. Dynamics of optical vortex solitons. *Opt. Commun.* **152**, 198–206 (1998).

[6] Kevrekidis, P. G., Frantzeskakis, D. J. & Carretero-González, R. *The defocusing nonlinear Schrödinger equation: from dark solitons and vortices to vortex rings* (SIAM, Philadelphia, 2015).

[7] Navarro, R. *et al.* Dynamics of a Few Corotating Vortices in Bose-Einstein Condensates. *Phys. Rev. Lett.* **110**, 225301 (2013).
