# Supplementary material for: Interactions and scattering of quantum vortices in a polariton fluid
Source: Nat Commun. 2018 Apr 13;9:1467. doi: 10.1038/s41467-018-03736-5 (PMC5899148; doi:10.1038/s41467-018-03736-5)
Supplement: Supplementary file 2 — Description of Additional Supplementary Files(PDF 246 kb) [file 41467_2018_3736_MOESM2_ESM.pdf]

Description of Additional Supplementary Files

File Name: Supplementary Movie 1
Description: Experimental dynamics depicting the short-range vortex scattering corresponding to the lowest total population P_1 in Fig. 2 of the main text. The amplitude and the phase of the polariton field are represented over a 100 x 100 μm^2 wide area and the time step is 0.5 ps. The video highlights the initial separation, the merging of the cores in the density field and their subsequent bounce-back.

File Name: Supplementary Movie 2
Description: Experimental dynamics depicting the short-range vortex scattering corresponding to the total population P_2 in Fig. 2 of the main text. The amplitude and the phase of the polariton field are represented over a 100 x 100 μm^2 wide area and the time step is 0.5 ps. The video highlights that the merging of the cores and their subsequent bounce-back are faster now with respect to the previous lesser power case.

File Name: Supplementary Movie 3
Description: Experimental dynamics depicting the short-range vortex scattering corresponding to the total population P_3 in Fig. 2 of the main text. The amplitude and the phase of the polariton field are represented over a 100 x 100 μm^2 wide area and the time step is 0.5 ps. The video highlights accelerated scattering dynamics and the induced background reshaping.

File Name: Supplementary Movie 4
Description: Experimental dynamics depicting the short-range vortex scattering corresponding to the largest total population P_4 in Fig. 2 of the main text. The amplitude and the phase of the polariton field are represented over a 100 x 100 μm^2 wide area and the time step is 0.5 ps. The video highlights such accelerated scattering dynamics that the merging cores is happening at very short times, and the induced background reshaping at later times is much more prominent than at lesser powers.

File Name: Supplementary Movie 5
Description: Numerical dynamics depicting the vortex scattering corresponding to the case described in the Supplementary Fig. 4. The density of the polariton field is represented over a 70 x 70 μm^2 wide area and the time step is 0.1 ps. The video highlights how the circular symmetry of the background density gradients is broken at short and intermediate time ranges, during the approaching of the vortices, and restored at longer times, after the scattering event.
